# Cause‐Specific Mortality and Prognostic Impact of Comorbidity in Japanese Patients With Chronic Lymphocytic Leukemia

**DOI:** 10.1002/cam4.70613

**Published:** 2025-01-28

**Authors:** Daisuke Ikeda, Takuya Nunomura, Tsuyoshi Muta, Kosei Matsue

**Affiliations:** ^1^ Division of Hematology/Oncology, Department of Internal Medicine Kameda Medical Center Chiba Japan; ^2^ Department of Hematology Hiroshima Red Cross Hospital and Atomic‐Bomb Survivors Hospital Hiroshima Japan

**Keywords:** cause of death and BTKi, CCI, CLL, comorbidities

## Abstract

**Background:**

Due to its rarity, there are very limited data available on the cause of death (COD) and its association with comorbidities in Japanese chronic lymphocytic leukemia (CLL) patients.

**Methods:**

To investigate the prevalence of comorbidities and their impact on cause‐specific mortality, we retrospectively reviewed 121 Japanese patients with CLL.

**Results:**

The median age was 69 years, with 47.9% having at least one comorbidity listed in the Charlson Comorbidity Index (CCI), and 12.4% were multimorbid. With a median follow‐up of 74 months, the 5‐ and 10‐year overall survival rates were 80.6% and 60.1%, respectively. Among the 44 deaths observed, CLL progression was the leading COD (38.6%), which together with infections and other malignancies accounted for nearly 80%. Patients with higher CCI risk categories had significantly higher 5‐year all‐cause mortality (CCI 1–2: 22.9% and ≥ 3: 31.4%) and non‐CLL‐specific mortality (CCI 1–2: 18.8% and ≥ 3: 31.4%) compared to those without (CCI 0: 12.6%, *p* = 0.005; 3.5%, *p* < 0.001, respectively), whereas CLL‐specific mortality was not influenced. On multivariate analysis, age and CCI retained a significant prognostic impact on all‐cause mortality (hazard ratio [HR] 1.08, *p* < 0.001 and HR 1.88, *p* = 0.004, respectively) and non‐CLL‐specific mortality (HR 1.12, *p* < 0.001 and HR 3.81, *p* < 0.001, respectively).

**Conclusions:**

Our study showed that CLL itself was the leading COD, and comorbidity burden was associated with non‐CLL‐specific deaths. This highlights the importance of better disease control and effective management of comorbidities.

## Graphical abstract

Due to its rarity, there are very limited data available on cause of death (COD) and its association with comorbidities in Japanese chronic lymphocytic leukemia (CLL) patients. Here, we report granular insight into treatment patterns, infectious disease burden, cause‐specific survival, and associated risk factors in a real‐world Japanese CLL cohort. Our study showed that CLL itself was the leading COD, and comorbidity burden was associated with non‐CLL‐specific deaths, highlighting the importance of better disease control and effective management of comorbidities.

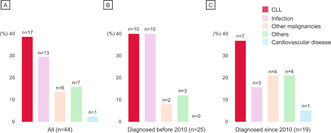

## 1 Introduction

Chronic lymphocytic leukemia (CLL) exhibits heterogeneous clinical behavior. Whereas one‐third of patients will never require active treatment, the prognosis for those in the highest‐risk group remains unsatisfactory in the 2020s [1]. Over the decades, understanding of the disease biology has expanded, resulting in the establishment of the CLL‐International Prognostic Index (CLL‐IPI) [2]. The CLL‐IPI integrates the status of *TP53* aberration and *IGHV* mutation into clinical data. This prognostic model can clearly stratify patient outcomes, which has been recently reassessed in the era of chemotherapy‐free targeted regimens such as Bruton's tyrosine kinase inhibitors (BTKi) [3]. However, the major caveat of this prognostic model is its difficulty in applying it to underrepresented populations outside of Western countries. For example, in Japan, CLL accounts for only 3% of total non‐Hodgkin lymphomas [4]. The rarity of CLL cases and limited access to the essential genetic tests constrain optimal patient management. Therefore, establishing a risk assessment independent of disease‐related factors may have greater clinical implications.

Since CLL primarily affects elderly adults and causes immunosuppression even in its early stages [5], comorbidities may be a significant determinant of outcomes. Although several Western groups aimed to dissect the interaction between comorbidities and outcomes [6, 7, 8, 9], related data have not been reported across Asian countries. In addition to the scarcity, the necessity of retrieving accurate information on cause‐specific mortalities in detail makes it challenging to conduct this type of study. To fill the knowledge gap, we conducted a multicenter retrospective observational study focusing on the cause of death (COD) and its association with baseline comorbidity burden in real‐world cohorts from two tertiary care centers in Japan.

## 2 Materials and Methods

### 2.1 Study Design, Patient, and Definition

We retrospectively analyzed patients with CLL/small lymphocytic leukemia (SLL) who were diagnosed between 1995 and 2022 at Kameda Medical Center and Hiroshima Red Cross Hospital and Atomic‐bomb Survivors Hospital and followed up at least once since 2005. Following clinical and laboratory data at diagnosis were collected from electronic chart review: age, gender, Eastern Cooperative Oncology Group performance status (ECOG PS), disease type (CLL or SLL), Rai and Binet stage, white blood cell counts, hemoglobin levels, platelet counts, albumin levels, creatinine levels, total bilirubin levels, and β2‐microglobulin levels (if available). The diagnosis of CLL/SLL was confirmed to be in accordance with the consensus guidelines of the International Workshop on Chronic Lymphocytic Leukemia (iwCLL) published in 2018 [10]. Data on cytogenetic abnormalities were limited but were collected for del(11q), trisomy 12, del(13q), and del(17p) when available. The anti‐CLL treatment was classified largely into (1) chemotherapy or chemoimmunotherapy, (2) anti‐CD20 antibody alone, (3) BTKi‐based therapy, and (4) venetoclax‐based therapy. For the primary objective of this study to focus on cause‐specific death of CLL, COD was categorized into the following: (1) CLL‐specific death and (2) non‐CLL‐specific death. CLL‐specific death was defined as death due to CLL progression, whereas non‐CLL‐specific death was defined as death due to causes other than CLL. The dataset was locked on December 31, 2022. This study was approved by each local institutional review board (approval number: 22‐123) and was conducted in accordance with the Declaration of Helsinki.

### 2.2 Comorbidity Assessment

Baseline comorbidities included in the Charlson Comorbidity Index (CCI) were reviewed. In detail, the CCI‐listed comorbidities are categorized into the following 19 conditions: myocardial infarction, chronic heart failure, peripheral vascular disease, cerebrovascular disease or transient ischemic attack, dementia, chronic obstructive pulmonary disease, connective tissue disease, peptic ulcer disease, liver disease with or without portal hypertension, diabetes mellitus requiring treatment with or without end‐organ damage, hemiplegia, moderate (creatinine levels > 3 mg/dL) or severe chronic kidney disease (on hemodialysis), localized or metastatic solid tumor, leukemia or lymphoma, and acquired immune deficiency syndrome. These comorbidities were scored based on their severity according to the original report proposed by Charlson et al. [11] CLL/SLL diagnosis was excluded for a hematological CCI score. Aligned with the original definition, patients were trichotomized into having a CCI score of 0, 1–2, or ≥ 3 as low‐, medium‐, and high‐risk comorbidity burden. Additionally, the presence of hypertension, dyslipidemia, and atrial fibrillation, which were also relevant during the treatment with BTKi, was recorded. Diabetes mellitus, hypertension, and dyslipidemia were considered comorbidities if the patients were receiving any medication for these conditions.

### 2.3 Statistical Analysis

The categorical and continuous variables were compared using Fisher's exact test and Mann–Whitney *U* tests, respectively. Overall survival (OS) was estimated by the Kaplan–Meier method with the log‐rank test. Time to next treatment (TTNT) as well as cumulative incidence of infectious disease hospitalization was estimated using Gray's test, with death treated as a competing event. In addition, cumulative incidence of death was analyzed separately for cause‐specific mortality, including all‐cause mortality, CLL‐specific mortality, and non‐CLL‐specific mortality, all using Gray's tests. For CLL‐specific mortality, deaths due to causes other than CLL progression were treated as competing events. Conversely, deaths due to CLL itself were analyzed as competing risks for non‐CLL‐specific mortality. The Cox‐proportional hazard model with the Fine‐Gray model was performed for univariate and multivariate analysis to identify risk factors for cause‐specific mortality. Age, CCI, and advanced stage (defined as Rai stage III–IV) were included as potential risk factors in this analysis. To evaluate the impact of BTKi usage at any treatment line on OS, we used a time‐dependent Cox‐proportional hazards regression model [12]. Because BTKi was not approved until May 2016 in Japan, time from diagnosis to first BTKi usage was considered a time‐varying covariate to avoid immortal time bias. A two‐sided *p*‐value of < 0.05 was considered statistically significant. All statistical analyses were performed using R version 1.4.1717 (R Foundation, Vienna, Austria).

## 3 Results

### 3.1 Baseline Patient Characteristics and Treatment Pattern

Overall, 121 patients with CLL/SLL were included, 81 (66.9%) of whom were diagnosed after 2010. Patient characteristics are shown in Table 1. The median age at diagnosis was 69 years old (interquartile range [IQR] 61–76), with the male predominance (77.7%). Representing indolent nature of the disease, most patients (97.5%) maintained their ECOG PS of ≦ 1. Almost half of patients (47.9%) had at least one CCI‐listed co‐existing disease, with 12.4% being multimorbid. By age group, there were no significant differences in the frequencies of patients with a CCI score of ≥ 1 among those aged < 60 (30.4%), 60–70 (44.7%), 70–80 (50.0%), and > 80 years (50.0%) (*p* = 0.291). Specifically, diabetes mellitus (17.8%), cerebrovascular disease (7.1%), and chronic liver disease (7.1%) were the top three frequent conditions (Table S1). Of particular interest in relation to BTKi usage, hypertension, cardiovascular disease, and atrial fibrillation were observed in 39.7% 15.7%, and 6.6% of patients, respectively. Among evaluable patients, cytogenetic abnormalities were identified as follows: del(13q) in 46.2%, trisomy12 in 20.0%, del(17p) in 14.0%, and del(11q) in 6.4%. The lower prevalence of del(11q) was consistent with a previous report from Asian countries [13, 14], which may reflect the geographical heterogeneity of cytogenetic characteristics. None of the patients was assessed for *TP53* and *IGHV* mutational status.

**TABLE 1:** Clinical characteristics of CLL/SLL patients in this analysis.

	Total patients (*n* = 121)
Patient factors	
Age, median (IQR)	69 (61–76)
Male, *n* (%)	94 (77.7)
ECOG PS > 1, *n* (%)	3 (2.5)
CCI except CLL/SLL, *n* (%)	
0	63 (52.1)
1	38 (31.4)
2	13 (10.7)
≥ 3	7 (5.8)
Disease factors, *n* (%)	
Disease subtype	
CLL	110 (90.9)
SLL	11 (9.1)
Time period at diagnosis	
Before 2010	40 (33.1)
Between 2010 and 2022	81 (66.9)
Rai staging (*n* = 110, only CLL)	
0–II	78 (70.9)
III–IV	26 (23.7)
Missing	6 (5.4)
Binet staging (*n* = 110, only CLL)	
A and B	84 (76.4)
C	20 (18.2)
Missing	6 (5.4)
Laboratory data, median (IQR)	
WBC, /μL	15,100 (11,250–20,400)
Hb, g/dL	12.9 (11.7–14.1)
Plt, /10^4^ μL	17.3 (13.2–21.7)
Alb, g/dL	4.2 (3.9–4.4)
Cre, mg/dL	0.76 (0.65–0.94)
TBil, mg/dL	0.65 (0.5–0.9)
β2MG, mg/dL (*n* = 90)	2.4 (1.7–3.4)
Cytogenetic abnormalities, *n* (%)	
del(11q) (data available *n* = 31)	2 (6.4)
Trisomy 12 (data available *n* = 70)	14 (20.0)
del(13q) (data available *n* = 39)	18 (46.2)
del(17p) (data available *n* = 57)	8 (14.0)

Abbreviations: Alb, albumin; CCI, Charlson Comorbidity Index; CLL, chronic lymphocytic leukemia; Cre, creatinine; ECOG PS, Eastern Cooperative Oncology Group Performance Status; Hb, hemoglobin; IQR, interquartile range; Plt, platelet; SLL, small lymphocytic lymphoma; TBil, total bilirubin; WBC, white blood cell; β2MG, beta 2 microglobulin.

In terms of treatment patterns, 46 (38.0%) patients remained treatment‐naive throughout the study period (Figure S1). The remaining 75 patients started CLL‐directed therapies with a median time to first treatment of 42 months (95% CI 31–66). The majority of these patients (*n* = 57 [76.0%]) received chemotherapy as first‐line treatment, most frequently using cyclophosphamide‐based (*n* = 36 [48.0%]), followed by fludarabine‐based (*n* = 12 [16.0%]), and other regimens (*n* = 9 [12.0%]). Thirty‐nine (52.0%) experienced at least one line of BTKi treatment, of whom 32 (82.1%) used ibrutinib only, four (10.2%) used acalabrutinib only, and the remaining three (7.7%) switched from ibrutinib to acalabrutinib. BTKi was selected for 17.3%, 31.1%, and 36.1% as first line, second line, and later lines (3/26 [11.5%] for third line and 14/21 [66.6%] for fourth line), respectively. The venetoclax‐based therapy was used only in three refractory patients (2.5%).

### 3.2 COD and Survival Analysis

**FIGURE 1:**
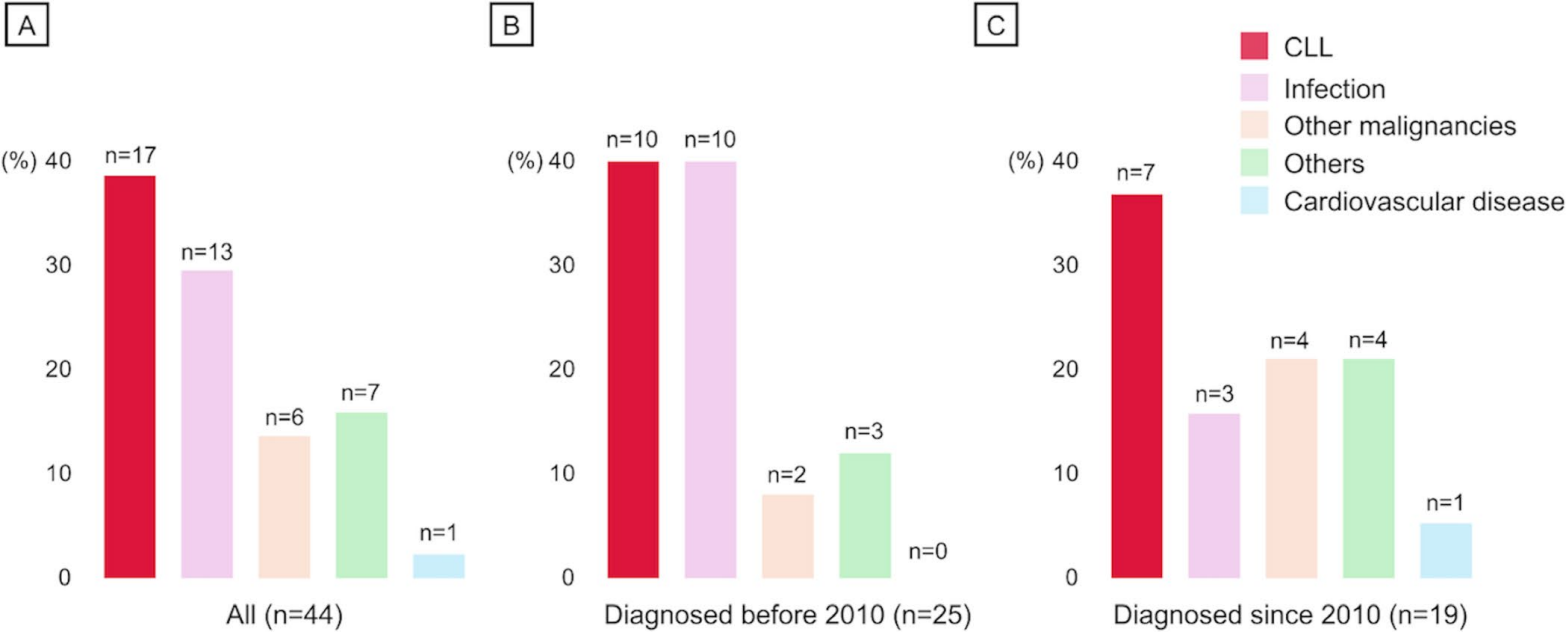
Bar chart illustrating the distribution of cause of death in this Japanese CLL cohort. (A) COD in all patients, (B) COD in patients diagnosed before 2010, and (C) since 2010. CLL, chronic lymphocytic leukemia; COD, cause of death.

During the median observation time of 74 months, the 5‐ and 10‐year OS were 80.6% (95% confidence interval [CI] 71.8–87.0) and 60.1% (95% CI 48.5–69.9), respectively (Figure S2). In total, 44 deaths occurred, with the 5‐ and 10‐year cumulative incidence of 19.3% (95% CI 11.8–36.8) and 38.0% (95% CI 27.4–48.5), respectively. Of note, CLL progression was the most frequent COD (17/44 [38.6%]) (Figure 1A). Moreover, infection and other malignancies, which are categorized as CLL‐related complications [6], accounted for approximately half of the mortality causes (19/44 [43.2%]). These two conditions were prevalent even in cases diagnosed after 2010 (Figure 1B,C). Among the 38 patients who were diagnosed after the first approval of ibrutinib in Japan, two deaths were observed: one died due to disease progression refractory to ibrutinib, and the other died of COVID‐19 during ibrutinib therapy. No cardiovascular mortality was observed during BTKi treatment in our cohort, with one case observed among non‐BTKi users.

**FIGURE 2:**
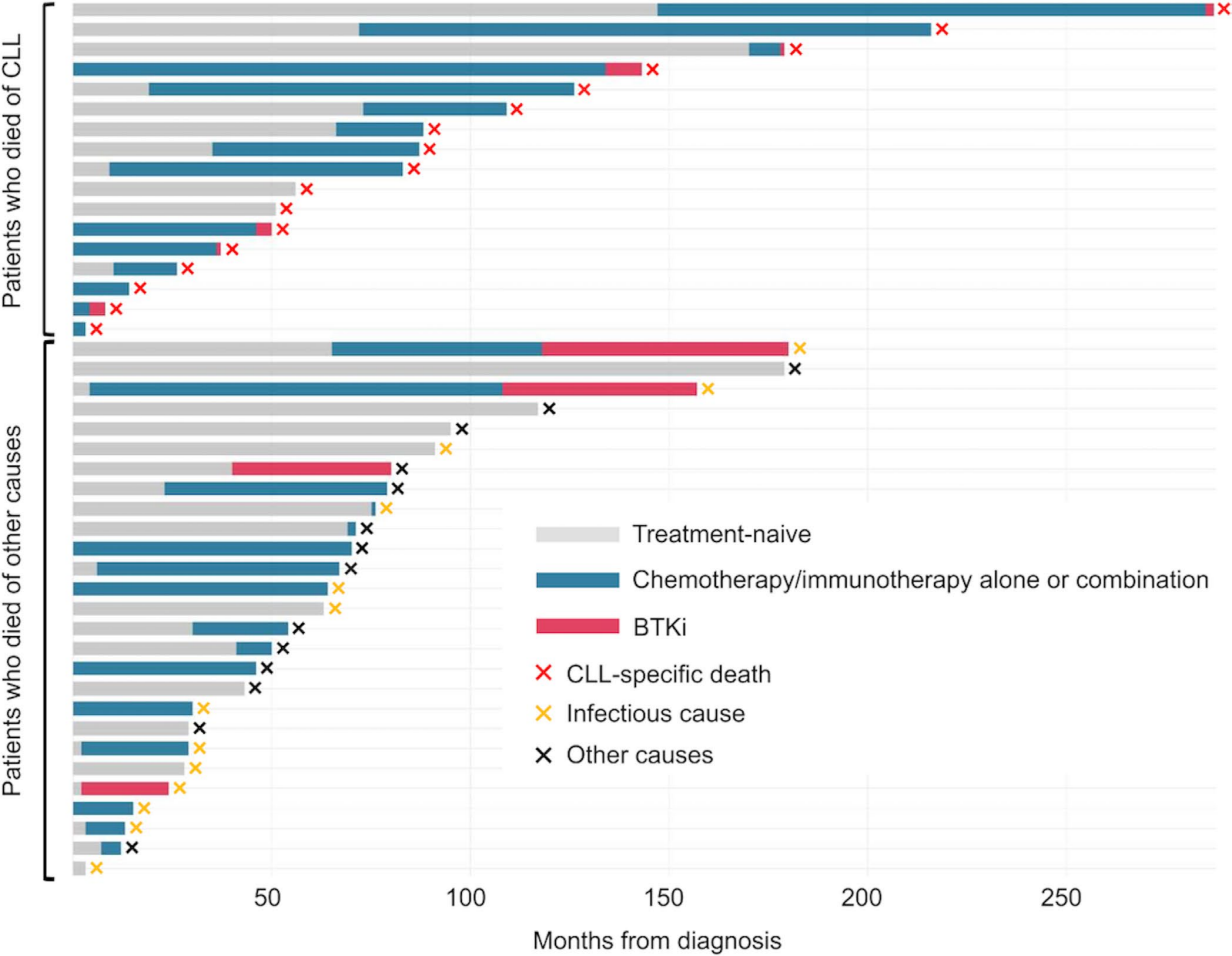
Swimmer plot demonstrating the clinical course of patients according to whether the cause of death was CLL progression. BTKi, Bruton's tyrosine kinase inhibitor; CLL, chronic lymphocytic leukemia.

The clinical courses of 44 patients who succumbed are shown in Figure 2. The age at diagnosis was significantly younger in CLL‐death patients (*n* = 17) than those in other causes (*n* = 27) (the median 68 [IQR 61–73] vs. 75 [IQR 68–81], *p* = 0.028). Despite the younger population advantage in the former group, this did not translate into survival advantage. Both groups had similar OS, with the 5‐year OS of 52.9% (95% CI: 27.6–73.0) in the CLL‐death group compared to 51.9% (95% CI 31.9–68.5) in the non‐CLL‐death group (*p* = 0.153) (Figure S3). Although the number of patients was small (*n* = 10), the duration of treatment with BTKi was significantly shorter in patients (*n* = 6) with death due to CLL progression than in those without (*n* = 4) (median of 2 months [IQR 1–3] vs. 32 months [IQR 23–42], *p* = 0.013), as illustrated in Figure 2. In addition, the time to first BTKi initiation was numerically longer in CLL‐death group than in non‐CLL‐death group (the median of 90 months [range 4–285] vs. 54 months [range 0–155], *p* = 0.336). These findings suggest the importance of durable disease control to improve survival and the potential benefit of longer BTKi exposure in this real‐world Japanese cohort.

### 3.3 Infectious Disease Hospitalization

**FIGURE 3:**
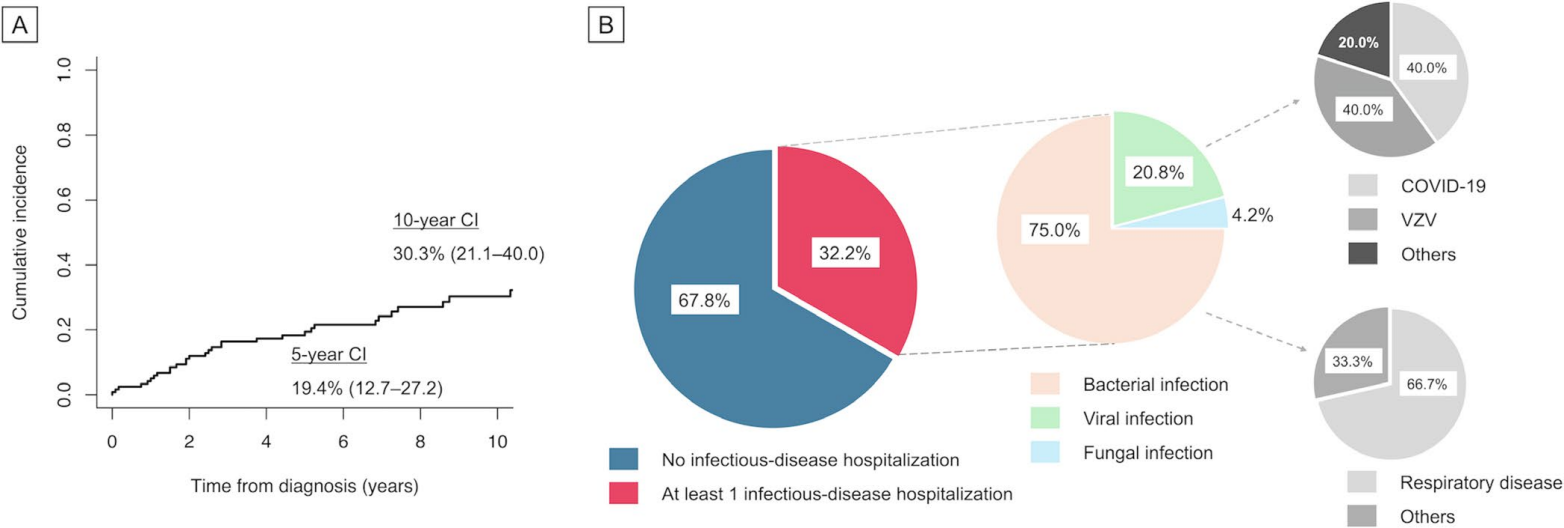
Cumulative incidence and details of infectious disease hospitalization. (A) Time to first infectious disease hospitalization from diagnosis. (B) Detailed reasons for hospitalization. CI, cumulative incidence; COVID‐19, coronavirus disease 2019; VZV, varicella‐zoster virus.

Given the frequent mortality due to infection, we next focused on the burden of infectious disease hospitalization in this cohort. The 5‐ and 10‐year cumulative incidence of the first infection event that required admission was estimated to be 19.4% (95% CI 12.7–27.2) and 30.3% (95% CI 21.1–40.0), respectively (Figure 3A). Thirty‐nine (32.2%) patients were hospitalized at least once, nearly half of whom (16/39 [41.0%]) experienced multiple admissions. Of total infections (*n* = 48), bacterial, viral, and fungal infections accounted for 36 (75.0%), 10 (20.8%), and two (4.2%) admissions (invasive candidiasis and disseminated cryptococcosis, respectively; Figure 3B). The case of cryptococcosis was observed within the first month after the ibrutinib initiation. This early onset is consistent with earlier reports showing that the vast majority of invasive fungal infections occur within the first 3 months of treatment [15]. As regards viral infections, herpes zoster (4/10 [40.0%], two of which were in a disseminated form) and COVID‐19 (4/10 [40.0%]) more frequently led to hospital admissions, whereas no cytomegalovirus‐associated hospitalizations were observed. One patient developed fatal COVID‐19 during the surge of the SARS‐CoV‐2 Delta variant while undergoing treatment with ibrutinib and achieving a partial response based on the iwCLL criteria. Respiratory tract was the most common site of bacterial infection (24/36, 66.7%), as expected. In detail, 13 of these 24 patients (54.2%) with a previous exposure to CLL therapies (10 undergoing treatment and three treated more than a year ago) developed respiratory bacterial infections, while 11 (45.8%) were treatment‐naive. Lastly, among the 39 BTKi‐treated patients, 13 (33.3%) developed an infection of any grade, with a median onset of 24 months (IQR 2–37) after BTKi initiation, which aligns with a real‐world prospective cohort from Italy (30.7%) [16].

### 3.4 Prognostic Factors for Cause‐Specific Mortality

**FIGURE 4:**
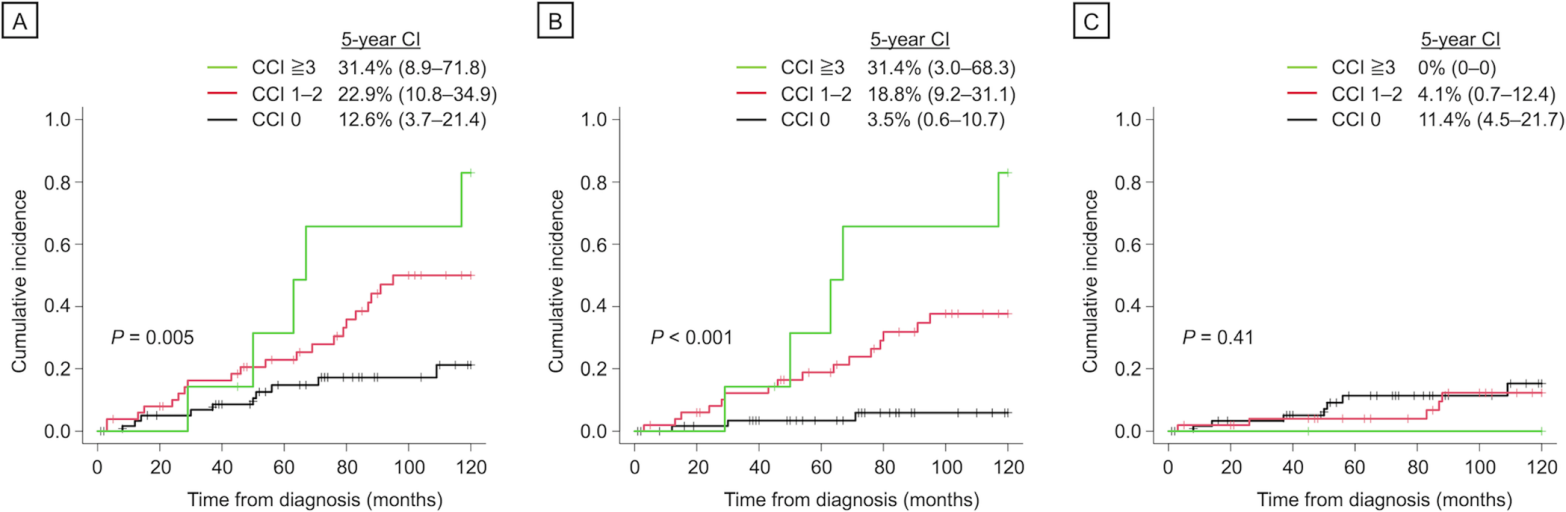
Five‐year cause‐specific mortality according to the CCI risk categories. (A) All‐cause mortality, (B) non‐CLL‐specific cause mortality, (C) CLL‐specific mortality. CCI, Charlson Comorbidity Index; CI, cumulative incidence; CLL, chronic lymphocytic leukemia.

Next, we aimed to identify predictors for cause‐specific mortality, particularly examining the impact of comorbidities. Patients with higher CCI scores had a significantly higher incidence of 5‐year all‐cause (CCI 1–2: 22.9% [95% CI 10.8–34.9]; CCI ≥ 3: 31.4% [95% CI 8.9–71.8]) and non‐CLL‐specific mortality (CCI 1–2: 18.8% [95% CI 9.2–31.1]; CCI ≥ 3: 31.4% [95% CI 3.0–68.3]) compared to those with a CCI score of 0 (12.6% [95% CI 3.7–21.4], *p* = 0.004; 3.5% [95% CI 0.6–10.7], *p* = 0.005 and *p* < 0.001, respectively) (Figure 4A,B). However, CLL‐specific mortality was not influenced by the presence of comorbid conditions (*p* = 0.41) (Figure 4C). After adjusting for the impact of age and disease stage, multivariate analysis confirmed the association of having comorbidities with a higher risk of non‐CLL‐specific mortality (hazard ratio [HR] 3.81, [95% CI 2.2–6.6], *p* < 0.001) (Table 2). Moreover, higher risk categories of CCI as well as elderly age at diagnosis retained its negative prognostic value for all‐cause mortality in the multivariate analysis (HR 1.88 [95% CI 1.21–1.93] and HR 1.08 [95% CI 1.03–1.12], *p* = 0.004 and *p* = 0.001, respectively). None of these variables significantly predicted the risks of CLL‐specific mortality.

**TABLE 2:** Univariate and multivariate analysis for mortality.

	Univariate analysis for all‐cause mortality			Multivariate analysis for all‐cause mortality		
	HR	95% CI	*p*	HR	95% CI	*p*
Age (as a continuous variable)	1.08	1.04–1.12	< 0.001	1.08	1.03–1.12	< 0.001
Male	0.98	0.53–1.78	0.94	—	—	—
Advanced stage^a^ (Rai III–IV)	3.2	1.65–6.19	< 0.001	1.91	0.95–3.86	0.068
CCI score (0, 1–2, or ≥ 3)	2.15	1.4–3.29	< 0.001	1.88	1.21–2.93	0.004

	Univariate analysis for CLL‐specific death			Multivariate analysis for CLL‐specific death		
	HR	95% CI	*p*	HR	95% CI	*p*
Age (as a continuous variable)	1.009	0.96–1.04	0.67	—	—	—
Male	0.41	0.16–1.08	0.073	—	—	—
Advanced stage^a^ (Rai III–IV)	0.94	0.23–3.87	0.94	—	—	—
CCI score (0, 1–2, or ≥ 3)	0.55	0.26–1.17	0.12	—	—	—

	Univariate analysis for non‐CLL‐specific death			Multivariate analysis for non‐CLL‐specific death		
	HR	95% CI	*p*	HR	95% CI	*p*
Age (as a continuous variable)	1.1	1.06–1.15	< 0.001	1.12	1.07–1.18	< 0.001
Male	1.77	0.77–4.09	0.18	—	—	—
Advanced stage^a^ (Rai III–IV)	4.37	2–9.53	< 0.001	1.79	0.8–3.98	0.15
CCI score (0, 1–2, or ≥ 3)	3.9	2.35–6.49	< 0.001	3.81	2.2–6.6	< 0.001

Abbreviations: CCI, Charlson Comorbidity Index; CI confidence interval; HR, hazard ratio.

^a^ The univariate and multivariate analyses, including advanced stage, were conducted for 110 patients with CLL who had available related data, not for those with SLL.

### 3.5 The Impact of BTKi Usage on OS and TTNT

Unexpectedly, the cox‐proportional hazard model dealing with time‐dependent covariates did not show survival benefit from treatment with BTKi at any line (HR 1.77 [95% CI 0.85–3.7], *p* = 0.125). This finding may be attributed to the short observation period from approval of BTKi in Japan (since 2016). Nevertheless, superior treatment durability with BTKi was observed compared to the conventional chemotherapy‐based treatment (Figure S4). For a second‐line treatment, BTKi showed a significantly longer TTNT than other treatments (2‐year probabilities of treatment change: 9.5% [95% CI 0.4–36.2] vs. 58.1% [95% CI 36.9–74.4], *p* = 0.002) (Figure S4A). In the frontline setting, the use of BTKi did not reach statistical significance in treatment durability (Figure S4B); however, the observed two cases of early treatment change within 2 years were both in‐class switching (i.e., ibrutinib to acalabrutinib): one due to an ibrutinib‐induced rash and the other due to physician's preference. Permitting these two cases, BTKi allowed for a significantly longer duration of use compared to other conventional therapies (2‐year probabilities of treatment change: 0% [95% CI not evaluable] vs. 42.2% [95% CI 29.6–54.2], *p* = 0.032) (Figure S4C).

## 4 Discussion

Due to its rarity, there are very limited data available on COD and its association with comorbidities in Japanese CLL patients. Here, we report granular insight into treatment patterns, infectious disease burden, cause‐specific survival, and associated risk factors in a real‐world Japanese CLL cohort. CLL itself was the greatest contributor to death, and when combined with infections and other malignancies, these causes accounted for approximately 80% of all deaths. Including cases that were not directly fatal, as many as 30% of patients were hospitalized at least once due to infectious complications within 10 years. Finally, we showed the negative prognostic impact of comorbidity burden on non‐CLL‐specific mortality independent of elderly age and advanced stage.

Historically, in Asian countries, research attention has been directed toward the distinct cytogenetic and molecular profiles of CLL compared to Western countries [7, 8, 9, 10, 11, 12]. For instance, several groups have reported the biased *IGHV* repertoires to the frequent *IGHV3* family usage with the underrepresentation of *IGHV1‐69*, which is the most common gene rearrangement observed in the West [14, 17, 18, 19]. The lower frequency of del(11q) (6.9%–12.5% and 6.4% in our study) has also been reported across different Asian countries and is considered to be ethnicity‐dependent [19, 20]. More recently, Takizawa et al. conducted a comprehensive review of the morphological and immunophenotypic characteristics, reporting a higher rate of mutated *IGHV*—reaching up to 80%—in Japanese CLL patients [14]. Although these findings have clearly contributed to a better understanding of the biological differences, they have not addressed another critical issue of how patient‐related factors such as age and comorbidity affect clinical outcomes in a manner similar to that seen in Western countries.

Besides the biological aspect, epidemiological trends have been investigated using registry data based on healthcare claims in Japan [21, 22, 23]. Takizawa et al. reported a longer time to first treatment from 2562 extracted patient data with a median of 1331 days (i.e., 43 months) [22], which was consistent with our current study (42 months). Regarding the prescription of BTKi, the administrative database study showed that only 18.6% of patients received ibrutinib in any treatment line among 2424 Japanese CLL patients during the period of 2013–2022 [23]. However, these reports are based on the Medical Data Vision (MDV) database, which aggregates medical bills and diagnostic procedures tied to disease names collected with consent from hospitals nationwide. Consequently, it is challenging to examine each patient in detail using medical records. Inferences must be made from the administered medications and hospitalization types to determine whether infections and complications are managed for prophylactic or therapeutic purposes.

Not only has there been no cause‐specific mortality analysis in Japan, but even simple survival analysis has only been reported for a small cohort of 29 CLL patients since the 2010s [24]. We reported the 5‐ and 10‐year OS rates of 80.6% and 60.1% with the median observation time of 74 months. This is very similar to the recent report from the Mayo Clinic using the large dataset of 1274 patients (5‐year OS of 83% and a 10‐year OS of 64%) [25]. More recently, a population‐based study in the Netherlands reported outcomes for 20,588 CLL patients diagnosed between 1996 and 2020, with 5‐ and 10‐year cumulative incidences of death at 27% and 43%, respectively [26]. This relatively higher mortality rate compared to our study (19.3% and 38.0%) may be attributed to the smaller proportion of patients diagnosed after 2010 in the Netherlands study (51.4% vs. 66.9% in the current study). In addition, we identified CLL progression as the most predominant COD (38.6%), which is consistent with reports from three out of four Western groups published between 2017 and 2021 (34.6%–46%) [6, 25, 27]. Rotbain et al. uniquely reported that infection was the most frequent COD (33.0%), regardless of comorbidity burden, with CLL progression following at 25.9% [7]. As discussed by the authors, the difference in rank of COD may be explained by the larger number of elderly individuals included in that study (the median age of 71 years vs. 63 years) [6, 25]. Together, our observations provide the first evidence that CLL itself, either directly or through CLL‐related complications, is a shared factor between Asian and Western populations determining prognosis.

Before the emergence of BTKi, more clinical trials of CLL tended to exclude patients with co‐existing conditions, despite it being a disease of the elderly, which resulted in a neglected comorbidity burden [28]. Since mid‐2010s, several groups investigated the interaction between comorbidities and survival in CLL. Although the association of comorbidities with inferior outcomes has been replicated in many studies [6, 7, 9, 27, 29, 30], some have reported conflicting results [8, 31, 32]. For instance, Gordon et al. showed that comorbidities did not significantly affect the outcomes of patients who received idelalisib therapy [31], whereas they also found that a high burden of comorbidities was associated with inferior OS in those treated with ibrutinib [33]. These discrepant results may largely stem from the highly heterogeneous study designs, particularly the different quantitative tools and definitions used to score the number and severity of comorbidities, such as the CCI versus the Cumulative Illness Rating Scale (CIRS). CIRS encompasses a broader range of organ systems beyond internal medicine and provides a more comprehensive index [34]; however, at the cost of detailed assessment, interrater agreement has been reported to be lower than that of CCI [35], and its applicability to real‐world cohorts outside of clinical trials seems to be limited. Given the lack of standardized and validated data, further real‐world investigation worldwide is needed to establish the role of comorbidities in CLL prognosis. In line with the largest prospective report from Mayo clinic (*n* = 1143) [6], multivariate analysis showed an independent association between comorbidities defined by CCI and higher risk of non‐CLL‐specific mortality, while CLL‐specific mortality was not affected. This observation reinforces the possibility that early detection and optimization of comorbid conditions in routine care can reduce the risk of potentially preventable CLL‐related complications and ultimately improve overall outcomes.

The strength of our study lies in the detailed data collection that captures even less severe cases of infectious disease hospitalization, which is not easily achieved in population‐based registry studies. On the other hand, several limitations should be considered. First, this report is based on patients from only two Japanese tertiary hospitals, which may not fully represent the broader Japanese population. The limitations also included the retrospective nature of the analysis and the relatively small number of patients, especially those treated with BTKi, despite the extended observation period. Although we found some evidences suggesting potential benefits of BTKi in real‐world settings, such as longer exposure to BTKi being less likely in the CLL‐death group and a tendency for patients on BTKi to have longer TTNT, an OS advantage was not observed using a Cox model with time‐dependent covariates, which may be related to the short observation period due to the delayed approval of BTKi in Japan. Finally, we missed some clinical data that could influence patient outcomes. Testing for the mutational status of *IGHV* was not commercially accessible, and information on *TP53* cytogenetic abnormalities was only partially available. The lack of association between comorbidity burden and CLL‐specific death, in turn, highlights the continuous need for appropriate prognostic testing in clinical practice [36] and a deeper understanding of disease biology beyond CLL‐IPI through emerging single‐cell and spatial technologies [37]. We also did not assess patient frailty beyond ECOG PS and CCI, which could play a significant role in determining treatment response and prognosis, particularly in elderly patients. Nevertheless, this study represents the first and largest series of CLL to assess cause‐specific mortality integrated with comorbidity burden reported in Japan to date.

In conclusion, we retrospectively analyzed the association between the cause‐specific mortality and comorbidities of Japanese patients with CLL. Given that CLL itself was the leading COD and comorbidity burden contributed to non‐CLL‐specific deaths, our study emphasizes the importance of disease control and managing comorbidities optimally.

## Author Contributions

**Daisuke Ikeda:** conceptualization (equal), data curation (equal), formal analysis (equal), investigation (equal), methodology (equal), writing – original draft (equal). **Takuya Nunomura:** conceptualization (equal), data curation (equal). **Tsuyoshi Muta:** conceptualization (equal), supervision (equal). **Kosei Matsue:** conceptualization (equal), funding acquisition (equal), supervision (equal), writing – original draft (equal), writing – review and editing (equal).

## Ethics Statement

This study was approved by each local institutional review board (approval number: 22‐123) and was conducted in accordance with the Declaration of Helsinki.

## Consent

Informed consent was obtained in the form of opt‐out on the website.

## Conflicts of Interest

K.M. received research fund from AstraZeneca. K.M. received honoraria from Janssen. Other authors have no conflicts of interest.

## Supporting information


**Figure S1.** Real‐world treatment patterns of Japanese CLL patients.

**Figure S2.** Overall survival of the entire patients included in this study.

**Figure S3.** Overall survival of 44 patients who died according to the cause of death.

**Figure S4.** Time to next treatment according to the types of treatment.

**Table S1.** Characteristics and frequencies of comorbidities.

## Data Availability

The data presented here will be provided upon reasonable request to the corresponding author (email: koseimatsue@gmail.com).
