# Supplementary material for: Cause‐Specific Mortality and Prognostic Impact of Comorbidity in Japanese Patients With Chronic Lymphocytic Leukemia
Source: Cancer Med. 2025 Jan 28;14(3):e70613. doi: 10.1002/cam4.70613 (PMC11773378; doi:10.1002/cam4.70613)
Supplement: Supplementary file 1 — Figure S1. Real‐world treatment patterns of Japanese CLL patients. [file CAM4-14-e70613-s005.pdf]

Fig. S1 Real-world treatment patterns of Japanese CLL patients

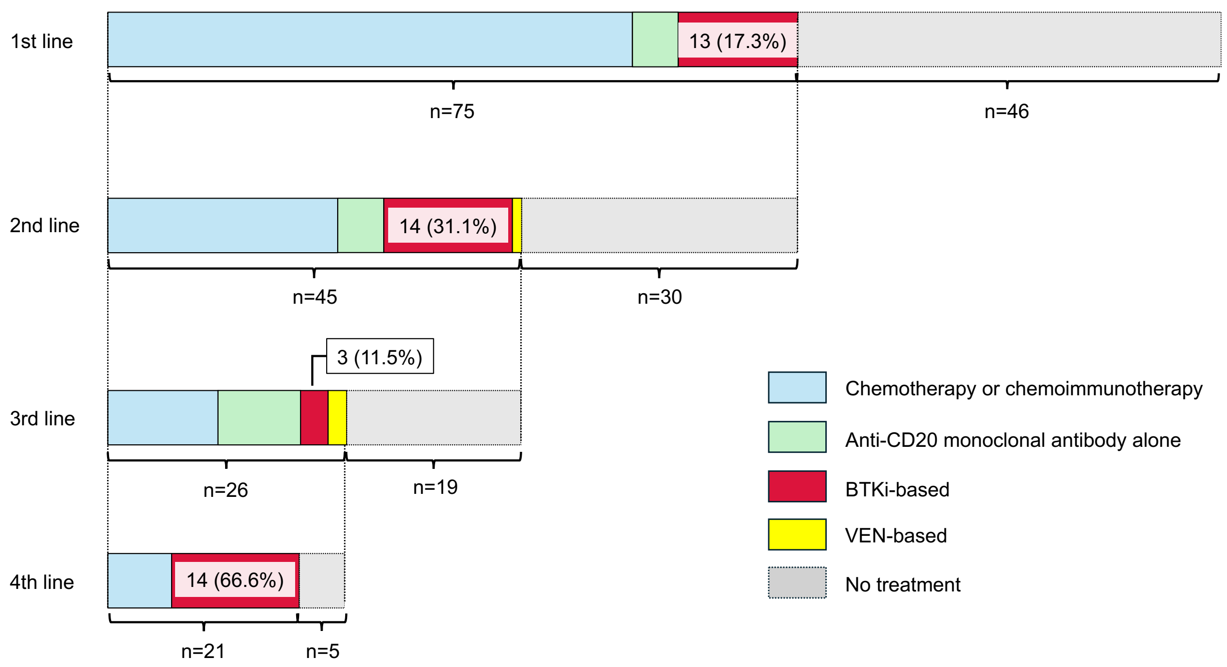

Abbreviations: Bruton’s tyrosine kinase inhibitor, Ven: Venetoclax
