# Supplementary material for: Cause‐Specific Mortality and Prognostic Impact of Comorbidity in Japanese Patients With Chronic Lymphocytic Leukemia
Source: Cancer Med. 2025 Jan 28;14(3):e70613. doi: 10.1002/cam4.70613 (PMC11773378; doi:10.1002/cam4.70613)
Supplement: Supplementary file 2 — Figure S2. Overall survival of the entire patients included in this study. [file CAM4-14-e70613-s004.pdf]

Fig. S2 Overall survival of the entire patients included in this study

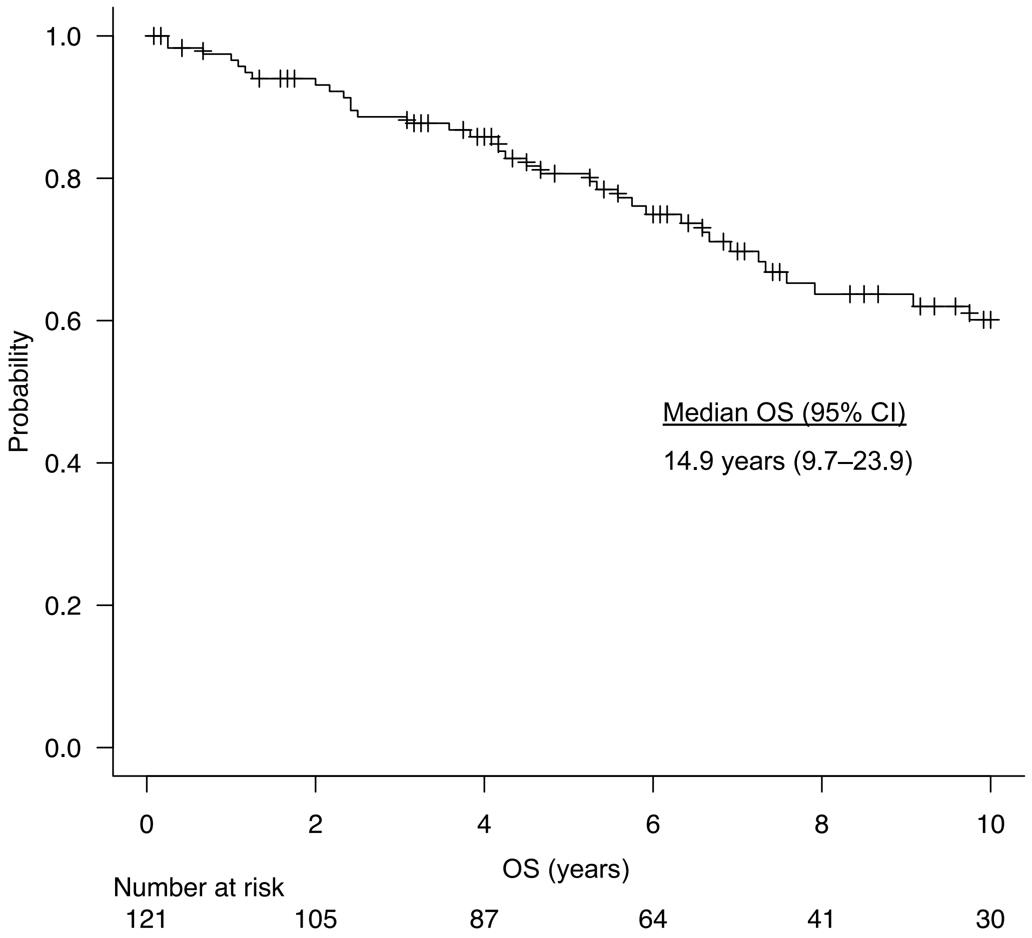

Abbreviation: OS; overall survival; CI, confidence interval.
