# Supplementary material for: Cause‐Specific Mortality and Prognostic Impact of Comorbidity in Japanese Patients With Chronic Lymphocytic Leukemia
Source: Cancer Med. 2025 Jan 28;14(3):e70613. doi: 10.1002/cam4.70613 (PMC11773378; doi:10.1002/cam4.70613)
Supplement: Supplementary file 3 — Figure S3. Overall survival of 44 patients who died according to the cause of death. [file CAM4-14-e70613-s002.pdf]

Fig. S3 Overall survival of 44 patients who died according to the cause of death

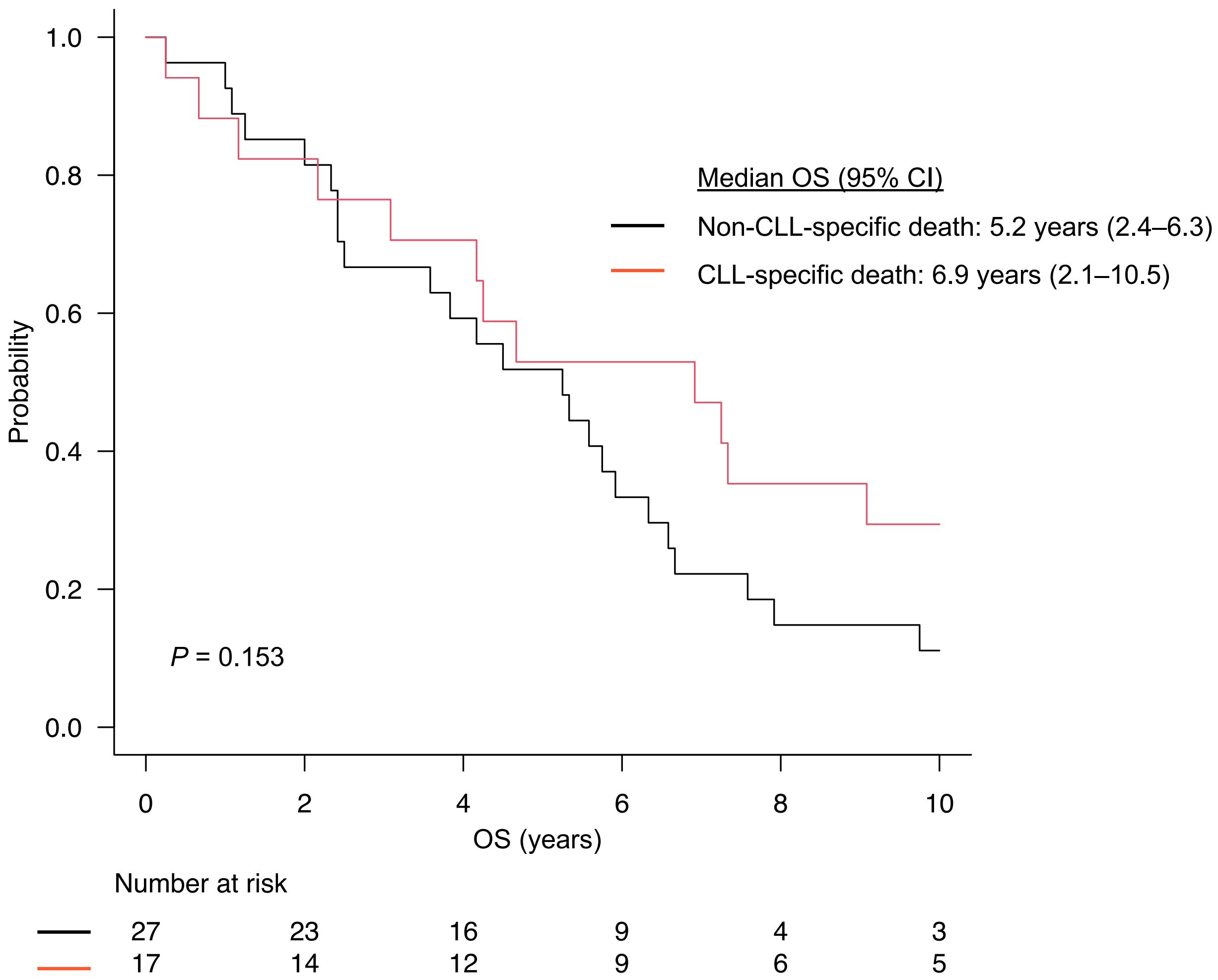

Abbreviation: OS; Overall survival, CI, confidence interval; CLL, chronic lymphocytic leukemia
