# Supplementary material for: Cause‐Specific Mortality and Prognostic Impact of Comorbidity in Japanese Patients With Chronic Lymphocytic Leukemia
Source: Cancer Med. 2025 Jan 28;14(3):e70613. doi: 10.1002/cam4.70613 (PMC11773378; doi:10.1002/cam4.70613)
Supplement: Supplementary file 4 — Figure S4. Time to next treatment according to the types of treatment. [file CAM4-14-e70613-s003.pdf]

Fig. S4 Time to next treatment according to the types of treatment

A) TTNT between 2nd and 3rd line treatment. B) TTNT between 1st and 2nd line treatment. The green circles indicate the two cases of switching from ibrutinib to acalabrutinib. C) TTNT between the 1st and 2nd line of treatment after allowing the two in-class BTKi switches.

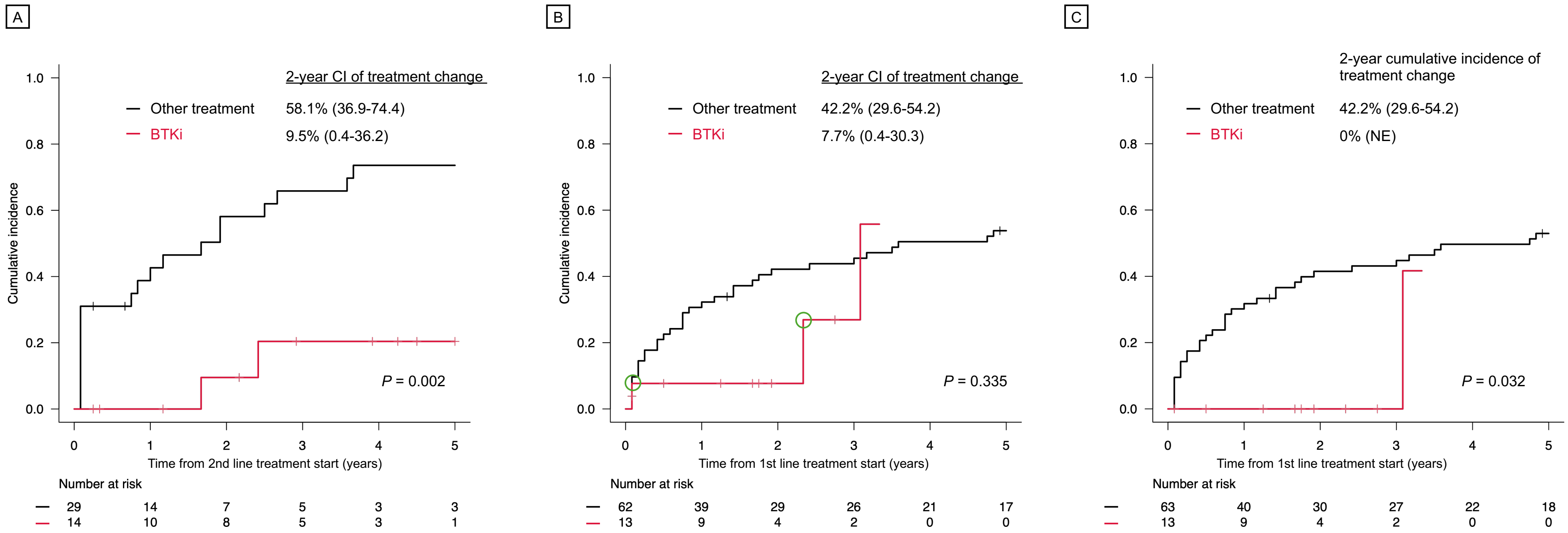

Abbreviations: TTNT, time to next treatment; BTKi, Bruton's tyrosine kinase inhibitor; CI, confidence interval; NE, not evaluable.
