# Supplementary material for: Cause‐Specific Mortality and Prognostic Impact of Comorbidity in Japanese Patients With Chronic Lymphocytic Leukemia
Source: Cancer Med. 2025 Jan 28;14(3):e70613. doi: 10.1002/cam4.70613 (PMC11773378; doi:10.1002/cam4.70613)
Supplement: Supplementary file 5 — Table S1. Characteristics and frequencies of comorbidities. [file CAM4-14-e70613-s001.docx]

**Table S1. Characteristics and frequencies of comorbidities**

| **Comorbidities listed in CCI** | **n (%)** |
| --- | --- |
| **Diabetes mellitus** | 20 (17.8) |
| **Stroke or transient ischemic attack** | 8 (7.1) |
| **Liver disease (mild to severe)** | 8 (7.1) |
| **Congestive heart failure** | 7 (6.2) |
| **Lung disease (mild)** | 7 (6.2) |
| **Solid tumors** | 7 (6.2) |
| **Myocardial infarction** | 5 (4.4) |
| **Collagen disease** | 5 (4.4) |
| **Peptic ulcer** | 4 (3.6) |
| **Peripheral artery disease** | 3 (2.7) |
| **Dementia** | 2 (1.8) |
| **Hematologic disease except CLL** | 2 (1.8) |
| **Hemiparesis** | 1 (0.9) |
| **AIDS** | 0 (0) |
| **Renal failure (moderate to severe)** | 0 (0) |
| **Number of CCI-listed comorbidities** | **n (%)** |
| **0** | 63 (52.1) |
| **1** | 43 (35.5) |
| **≧2** | 15 (12.4) |
| **Other comorbidities** | **n (%)** |
| **Hypertension** | 48 (39.7) |
| **Dyslipidemia** | 22 (18.2) |
| **Cardiovascular disease*** | 19 (15.7) |
| **Atrial fibrillation** | 8 (6.6) |

Abbreviations: AIDS; acquired immunodeficiency syndrome, CCI; Charlson Comorbidity Index

* Cardiovascular disease in this category includes CCI-listed cardiovascular morbidities such as stroke, transient ischemic attack, heart failure, myocardial infarction and peripheral artery disease
